# Supplementary material for: Nor-24-homoscalaranes, Neutrophilic Inflammatory Mediators from the Marine Sponge Lendenfeldia sp
Source: Pharmaceuticals (Basel). 2023 Sep 6;16(9):1258. doi: 10.3390/ph16091258 (PMC10537518; doi:10.3390/ph16091258)
Supplement: Supplementary file 1 [file pharmaceuticals-16-01258-s001.zip › pharmaceuticals-2496093-supplementary.pdf]

SUPPLEMENTARY MATERIAL

Nor-24-homoscalaranes, Neutrophilic Inflammatory Mediators from the Marine Sponge *Lendenfeldia* sp.

Contents

S1. IR spectrum of compound 1 3

S2. ESIMS spectrum of compound 1 3

S3. HRESIMS spectrum of compound 1 4

S4. ^{1}H NMR spectrum (400 MHz) of compound 1 in $CDCl_3$. 4

S5. ^{13}C NMR spectrum (100 MHz) of compound 1 in $CDCl_3$. 5

S6. HSQC spectrum of compound 1 in $CDCl_3$. 6

S7. HMBC spectrum of compound 1 in $CDCl_3$. 6

S8. ^{1}H-^{1}H COSY spectrum of compound 1 in $CDCl_3$. 7

S9. NOESY spectrum of compound 1 in $CDCl_3$. 7

S10. IR spectrum of compound 2 8

S11. ESIMS spectrum of compound 2. 8

S12. HRESIMS spectrum of compound 2 9

S13. ^{1}H NMR spectrum (600 MHz) of compound 2 in $CDCl_3$. 9

S14. ^{13}C NMR spectrum (100 MHz) of compound 2 in $CDCl_3$. 10

S15. HSQC spectrum of compound 2 in $CDCl_3$. 10

S16. HMBC spectrum of compound 2 in $CDCl_3$. 11

S17. ^1H-^1H COSY spectrum of compound 2 in $CDCl_3$. 11

S18. NOESY spectrum of compound 2 in $CDCl_3$. 12

S19. IR spectrum of compound 3. 13

S20. ESIMS spectrum of compound 3. 13

S21. HRESIMS spectrum of compound 3. 14

S22. ^1H NMR spectrum (400 MHz) of compound 3 in $CDCl_3$. 14

S23. ^{13}C NMR spectrum (100 MHz) of compound 3 in $CDCl_3$. 15

S24. DEPT spectrum of compound 3 in $CDCl_3$. 15

S25. HSQC spectrum of compound 3 in $CDCl_3$. 16

S26. HMBC spectrum of compound 3 in $CDCl_3$. 16

S27. 1H-1H COSY spectrum of compound 3 in $CDCl_3$. 17

S28. NOESY spectrum of compound 3 in $CDCl_3$. 17

S29. IR spectrum of compound 4. 19

S30. ESIMS spectrum of compound 4. 20

S31. HRESIMS spectrum of compound 4. 20

S32. ^1H NMR spectrum (600 MHz) of compound 4 in $CDCl_3$. 21

S33. ^{13}C NMR spectrum (150 MHz) of compound 4 in $CDCl_3$. 21

S34. HSQC spectrum of compound 4 in $CDCl_3$. 21

S35. HMBC spectrum of compound 4 in CDCl₃. 22

S36. ^{1}H-^{1}H COSY spectrum of compound 4 in CDCl₃. 22

S37. NOESY spectrum of compound 4 in CDCl₃. 23

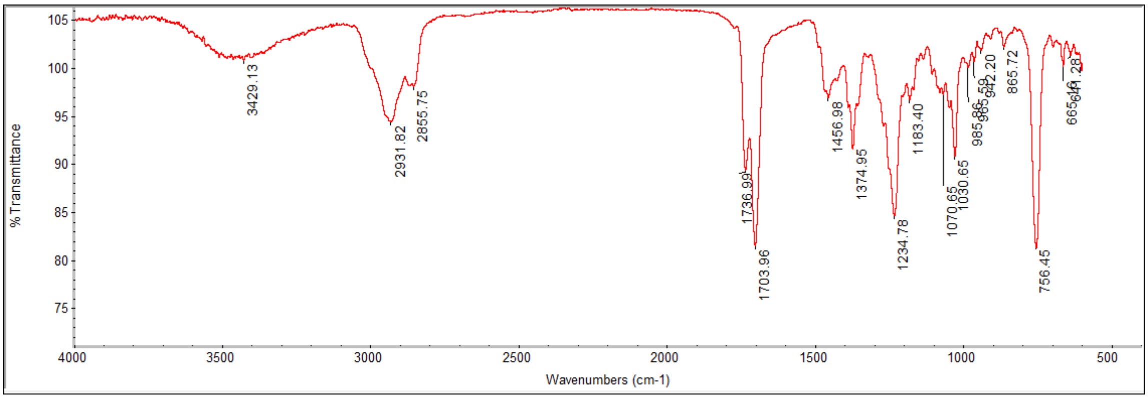

S1. IR spectrum of compound **1**.

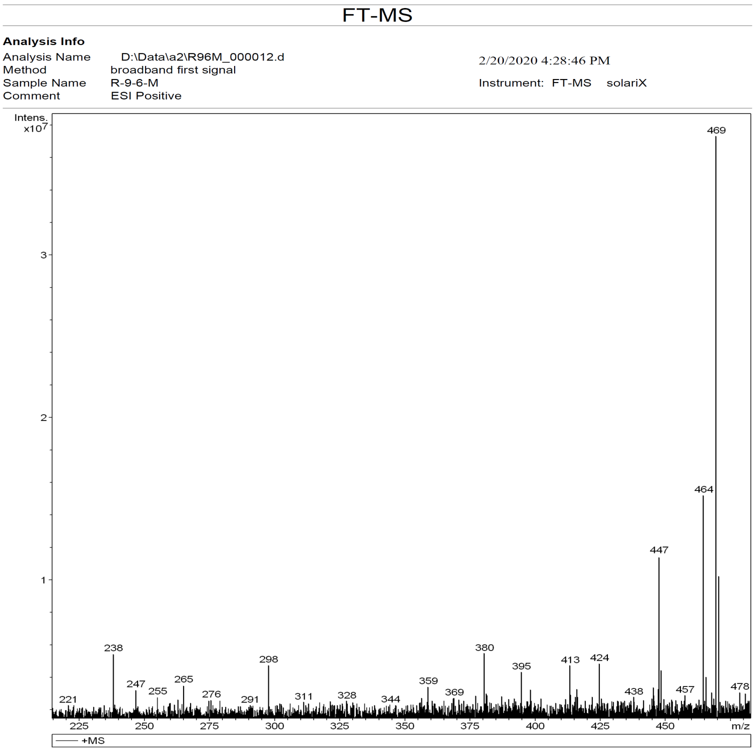

S2. ESIMS spectrum of compound **1**.

Mass Spectrum SmartFormula Report

Analysis Info

Analysis Name	D:\Data\a2\R96M_000011.d	2/20/2020 4:28:02 PM
Method	broadband first signal	Operator: YU HSIAO-CHING
Sample Name	R-9-6-M	Instrument: BRUKER FT-MS solariX
Comment	ESI Positive	

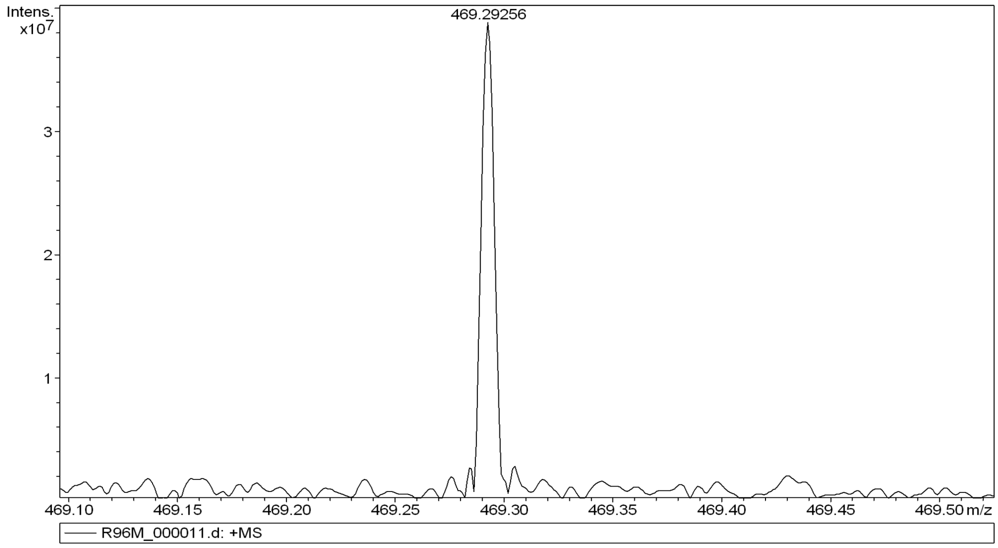

Meas. m/z	#	Formula	Score	m/z	err [mDa]	err [ppm]	mSigma	rdb	e⁻ Conf	N-Rule
469.29256	1	C 27 H 42 Na O 5	100.00	469.29245	-0.12	-0.25	32.1	6.5	even	ok

S3. HRESIMS spectrum of compound **1**.

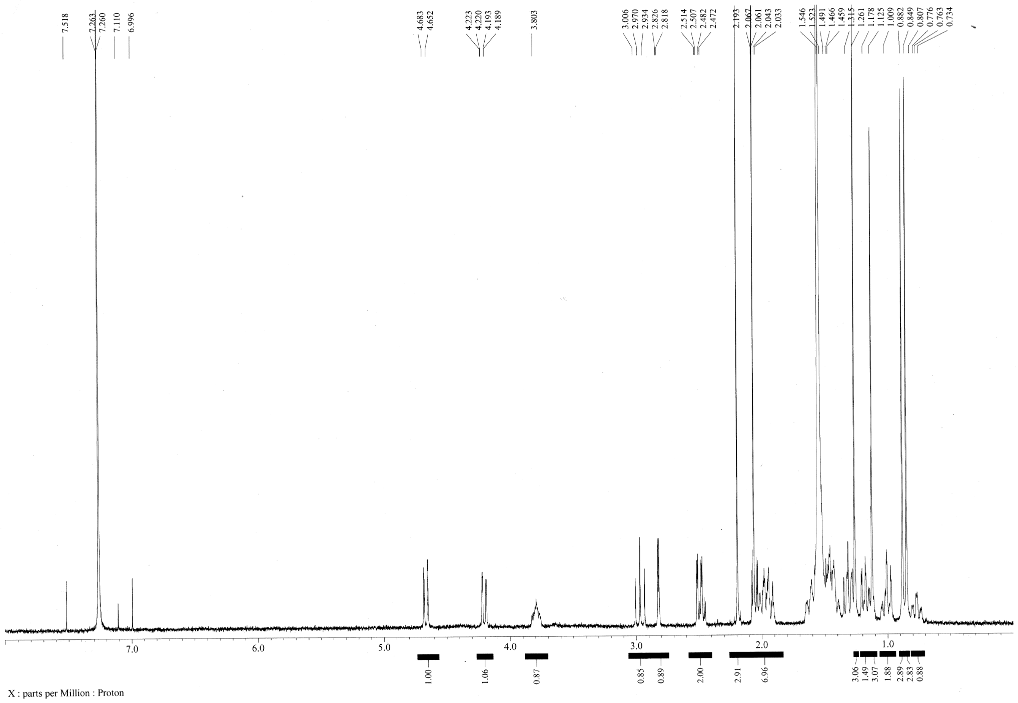

S4. ^{1}H NMR spectrum (400 MHz) of compound **1** in $CDCl_3$.

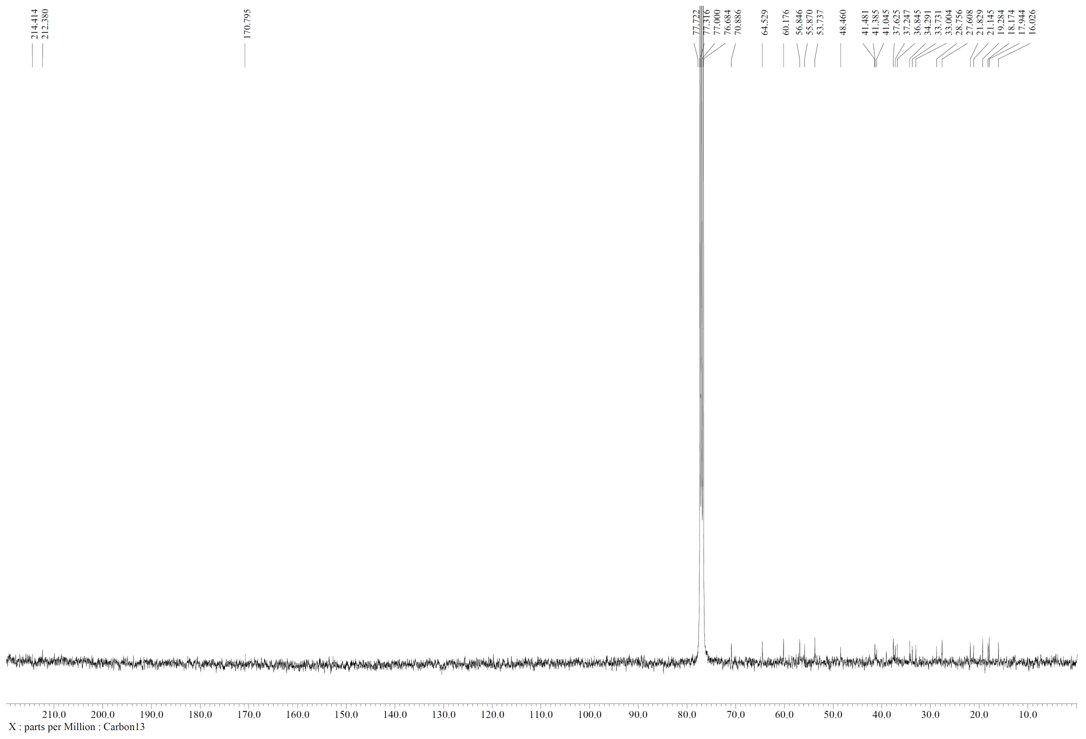

S5. ^{13}C NMR spectrum (100 MHz) of compound **1** in $CDCl_3$.

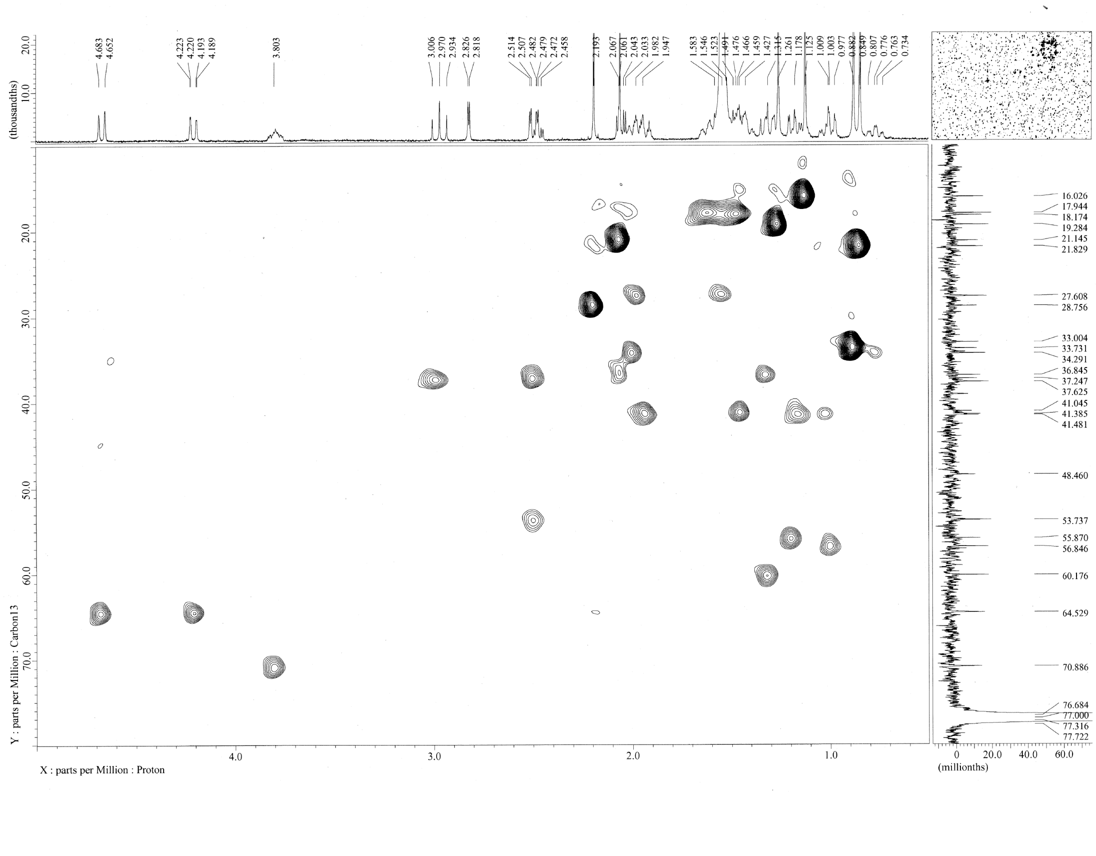

S6. HSQC spectrum of compound **1** in $CDCl_3$.

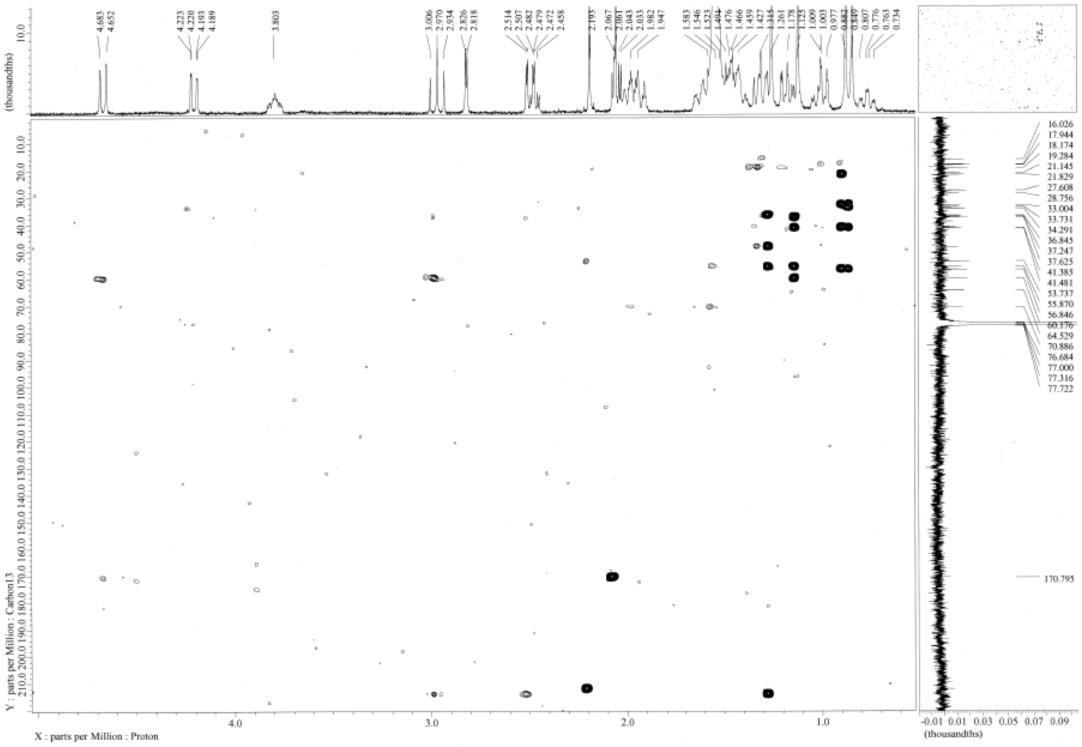

S7. HMBC spectrum of compound **1** in $CDCl_3$.

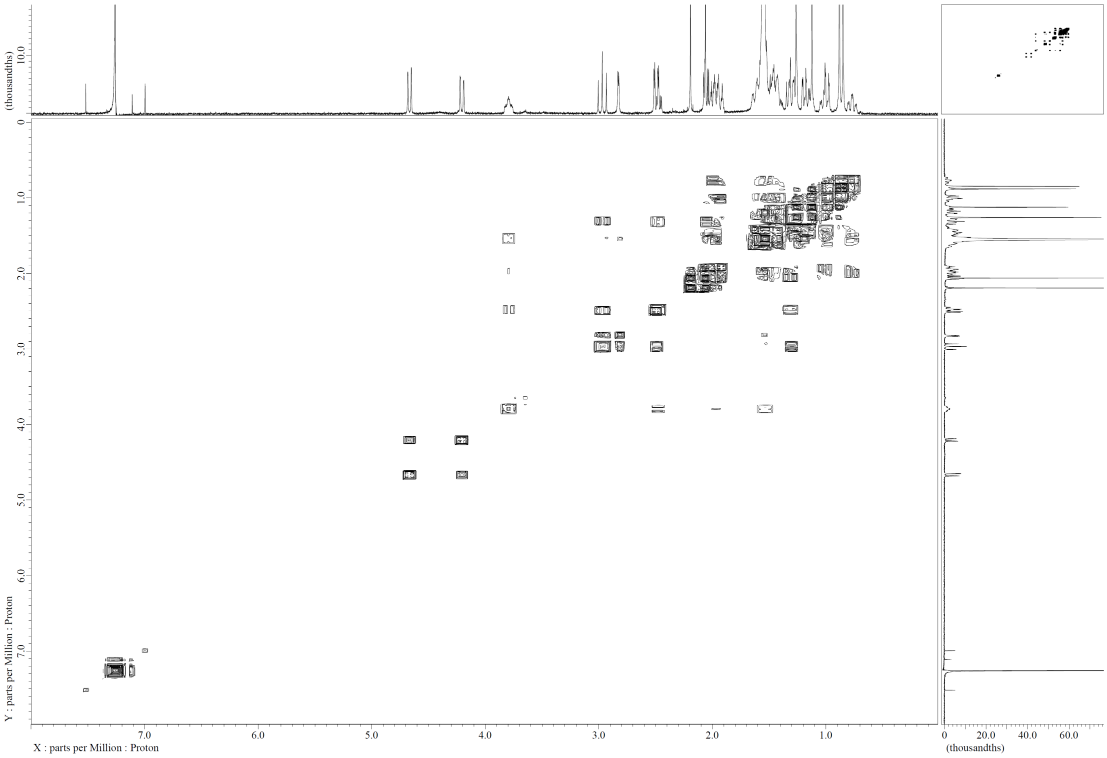

S8. ^{1}H-^{1}H COSY spectrum of compound **1** in $CDCl_3$.

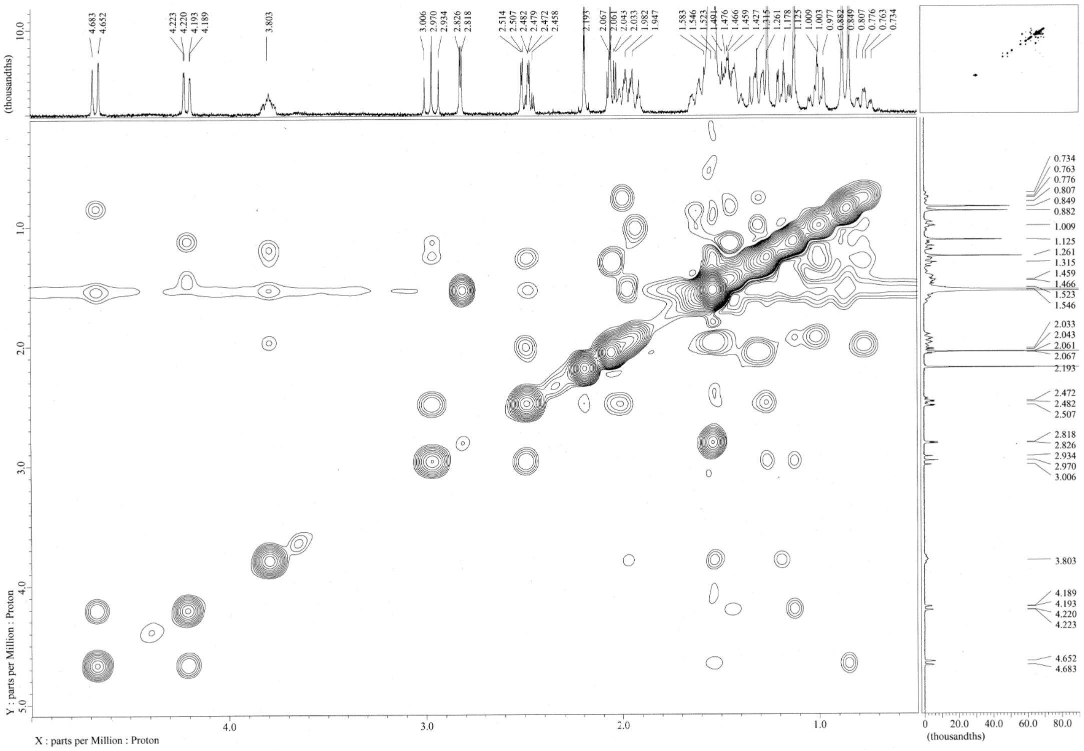

S9. NOESY spectrum of compound **1** in $CDCl_3$.

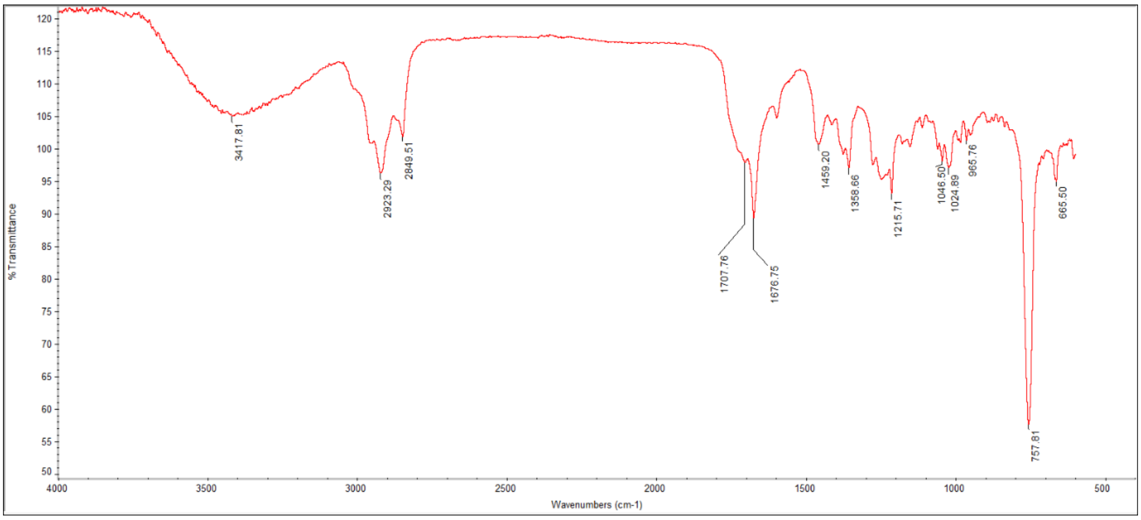

S10. IR spectrum of compound **2**.

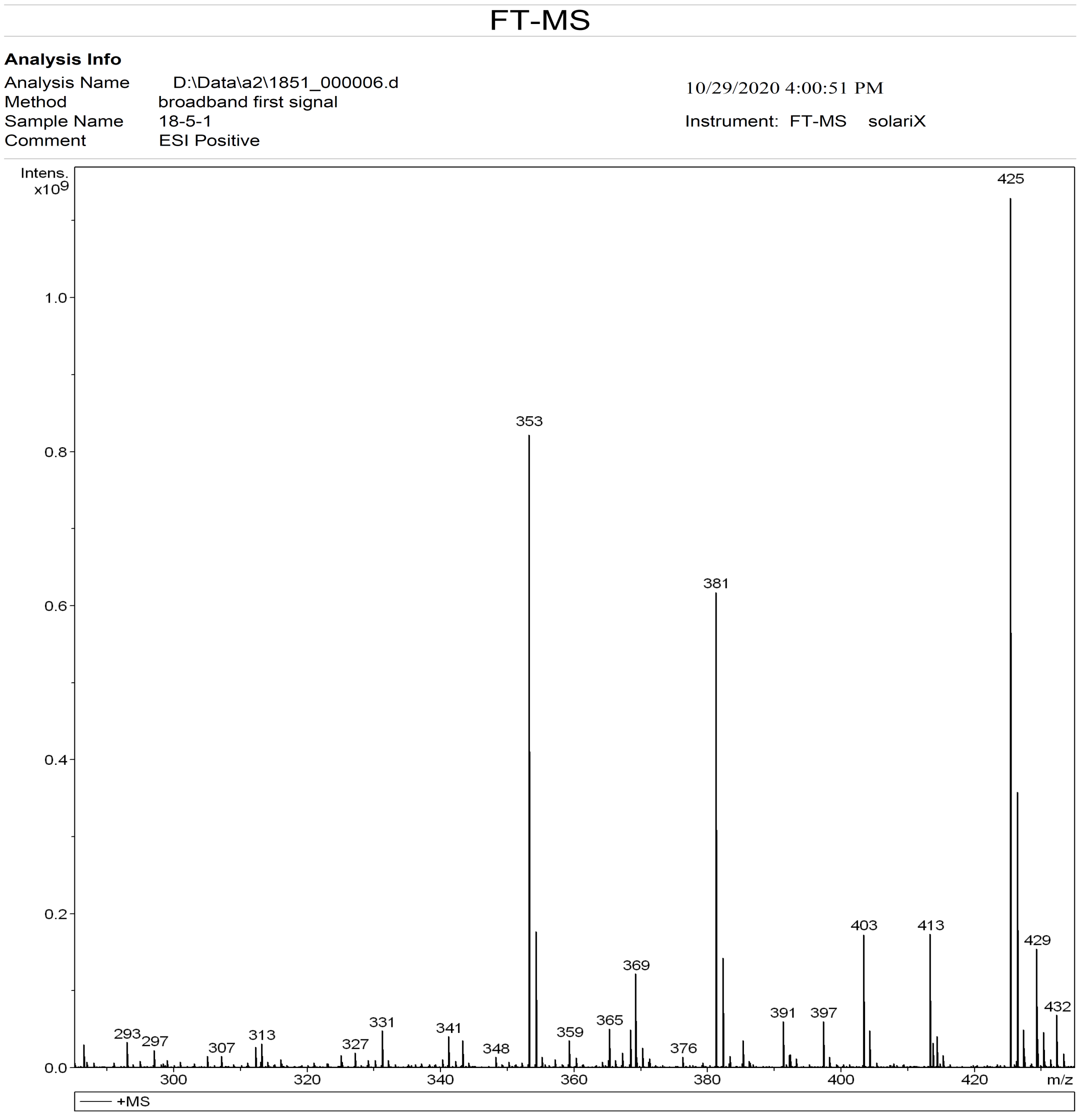

S11. ESIMS spectrum of compound **2**.

Mass Spectrum SmartFormula Report

Analysis Info

Analysis Name	D:\Data\a2\1851_000007.d	11/12/2020 11:48:17 AM
Method	broadband first signal	Operator: YU HSIAO-CHING
Sample Name	18-5-1	Instrument: BRUKER FT-MS solariX
Comment	ESI Positive	

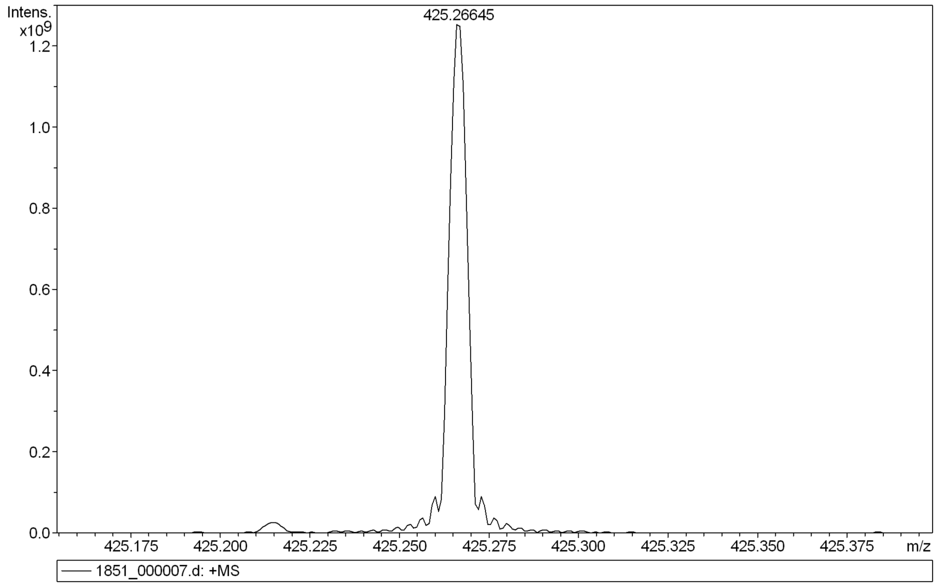

Meas. m/z	#	Formula	Score	m/z	err [mDa]	err [ppm]	mSigma	rdb	e⁻ Conf	N-Rule
425.26645	1	C 25 H 38 Na O 4	100.00	425.26623	-0.22	-0.52	40.1	6.5	even	ok

S12. HRESIMS spectrum of compound **2**.

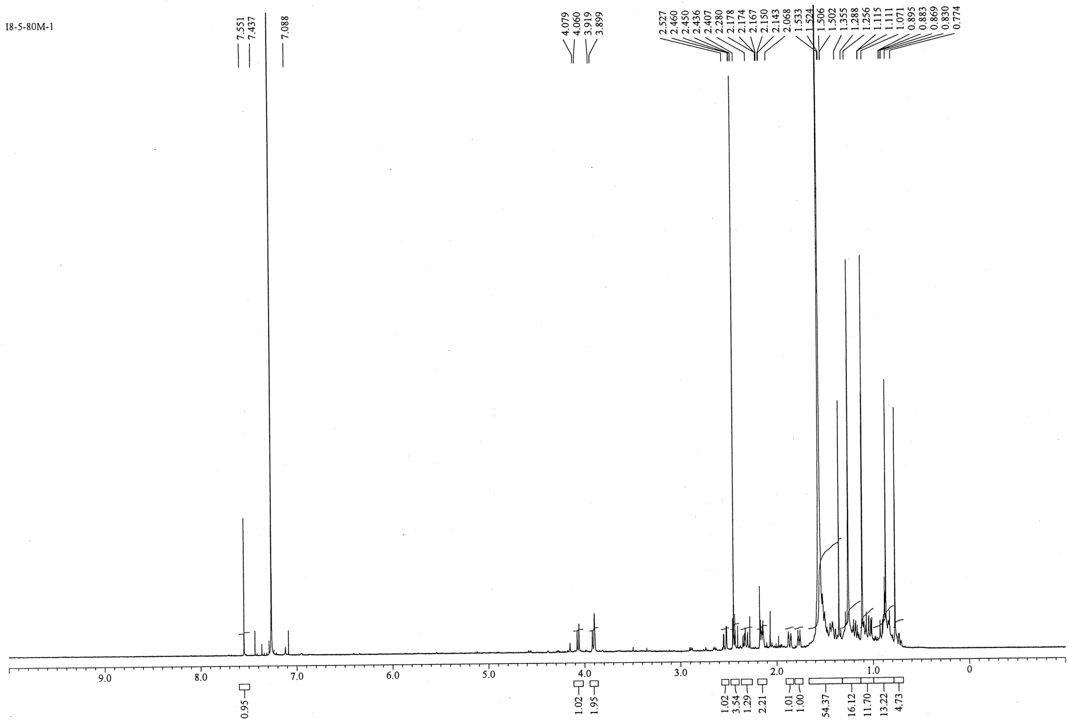

S13. ^{1}H NMR spectrum (600 MHz) of compound **2** in $CDCl_3$.

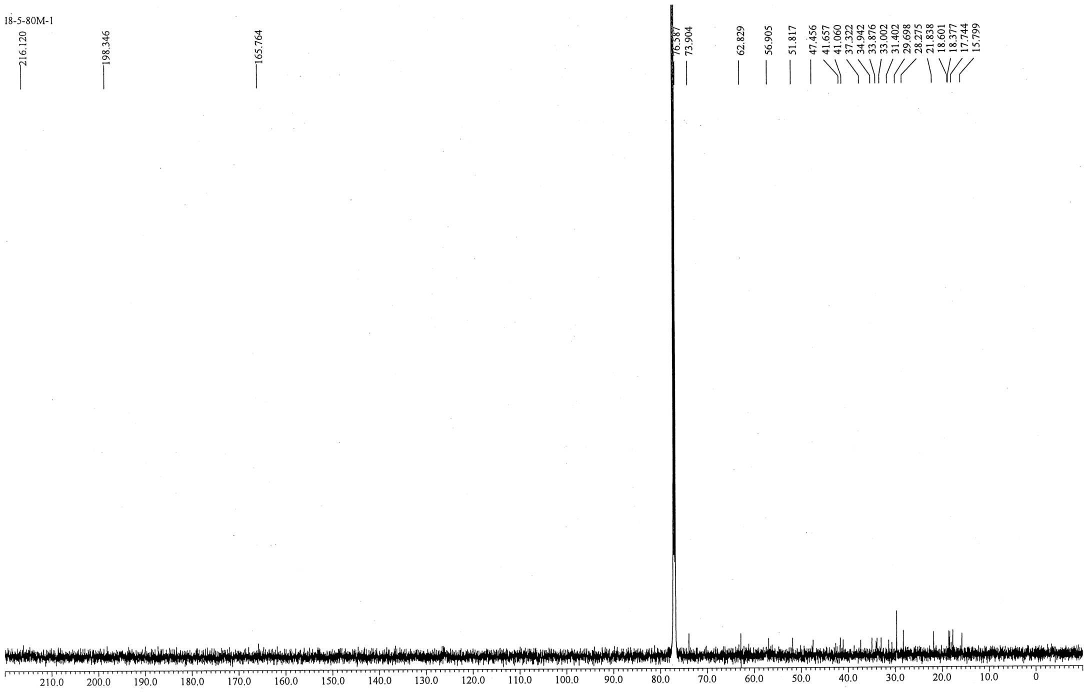

S14. ^{13}C NMR spectrum (100 MHz) of compound **2** in $CDCl_3$.

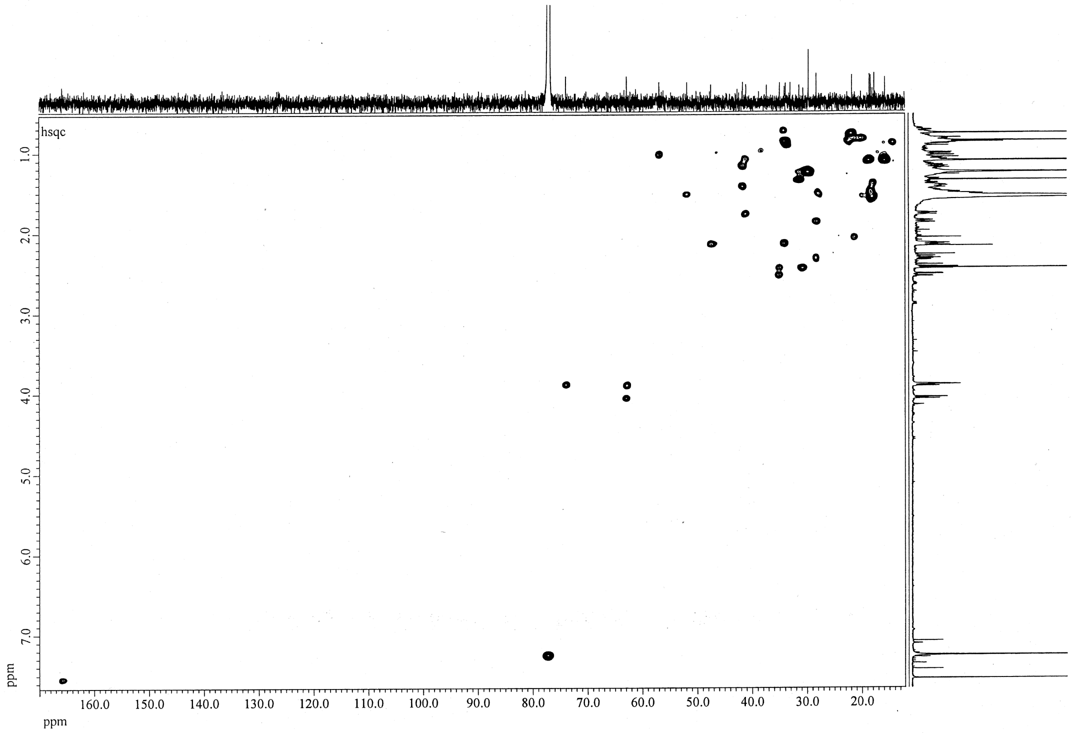

S15. HSQC spectrum of compound **2** in $CDCl_3$.

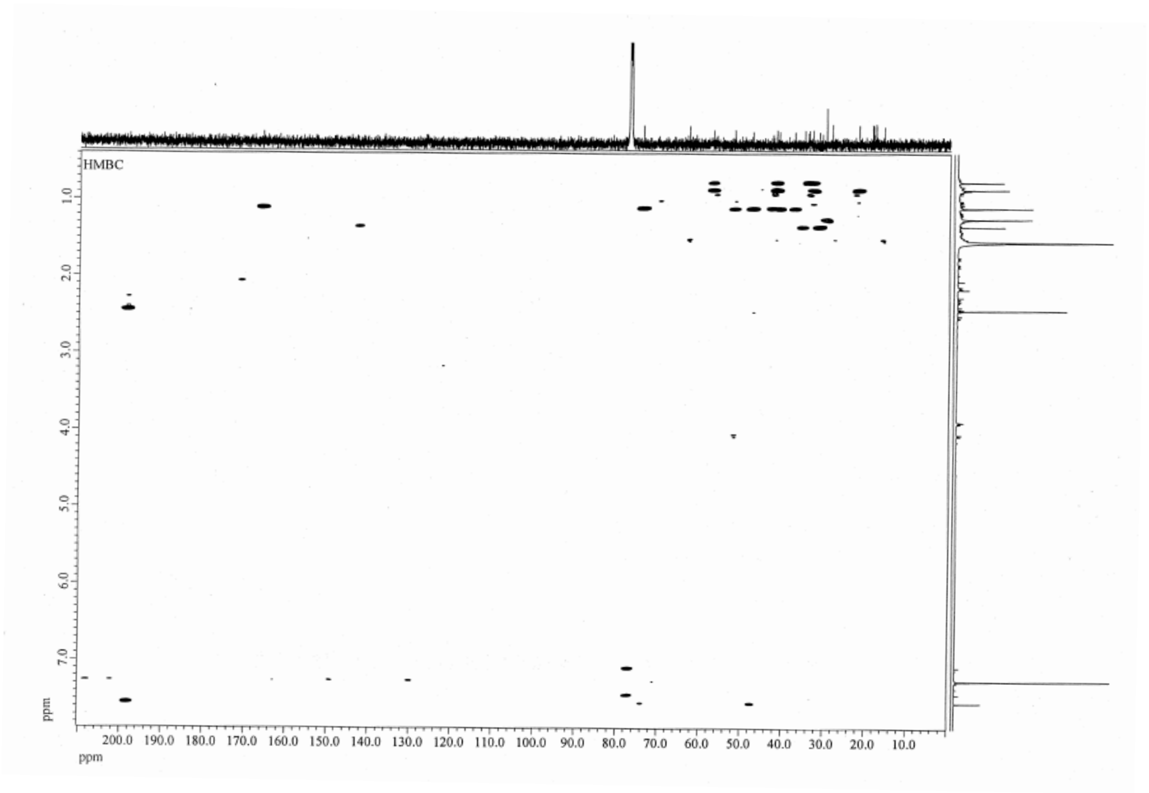

S16. HMBC spectrum of compound **2** in $CDCl_3$.

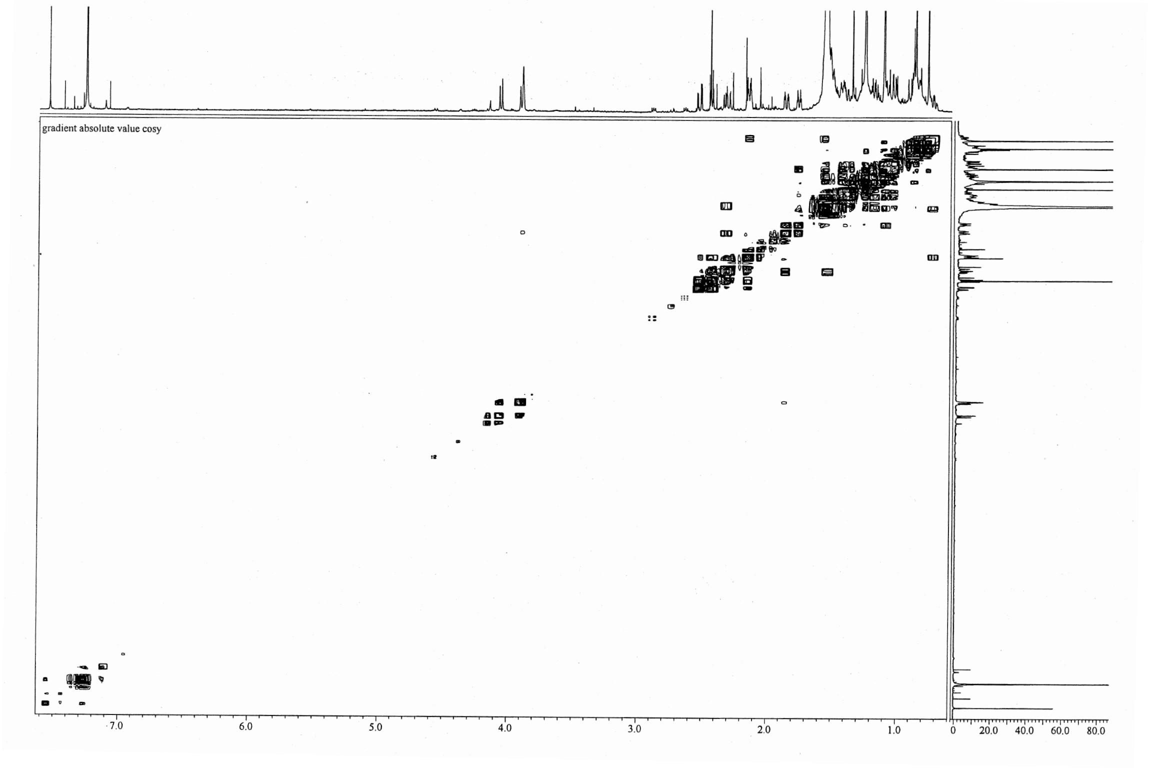

S17. ^{1}H-^{1}H COSY spectrum of compound **2** in $CDCl_3$.

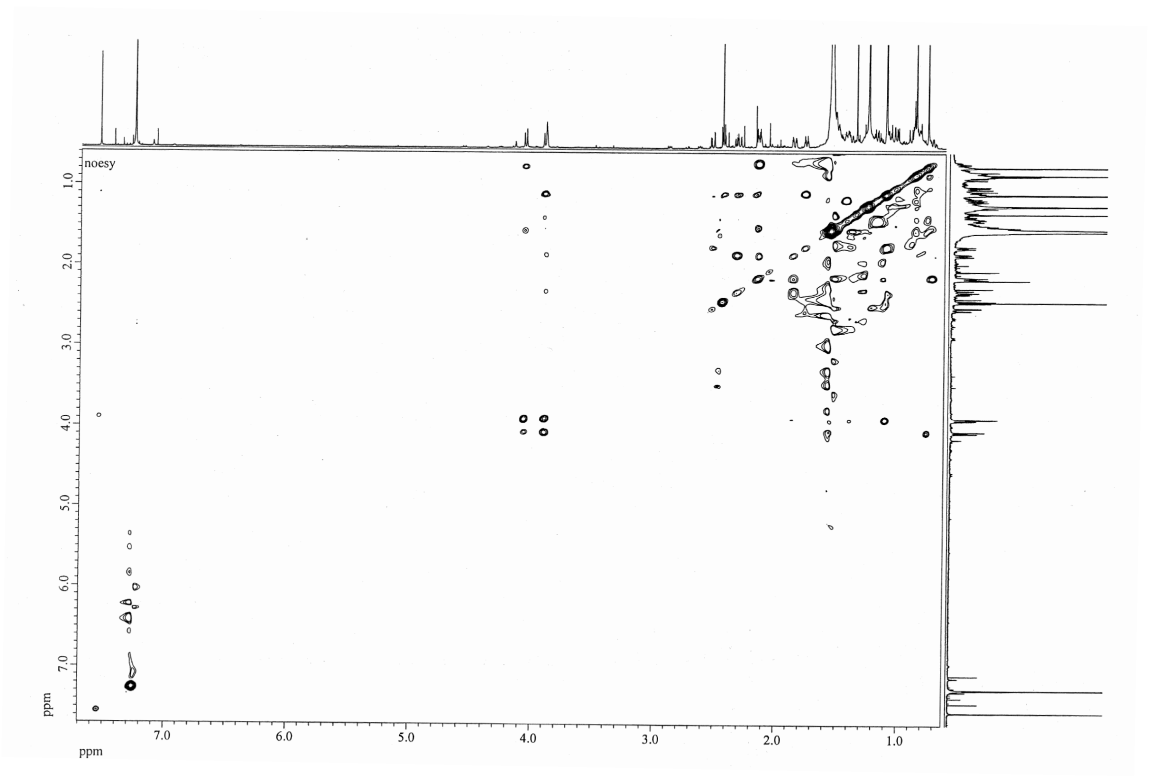

S18. NOESY spectrum of compound **2** in $CDCl_3$.

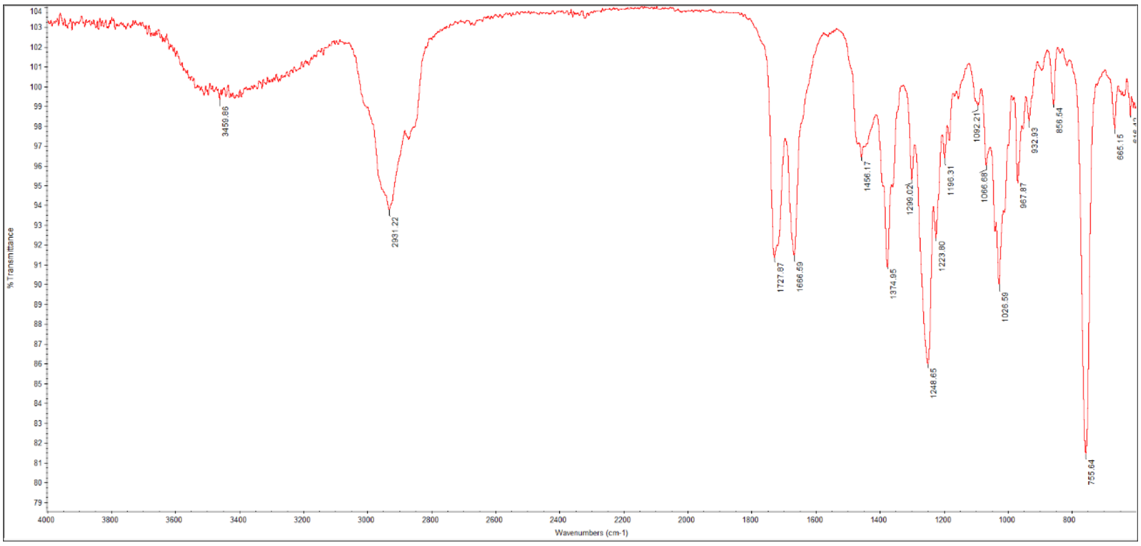

S19. IR spectrum of compound **3**.

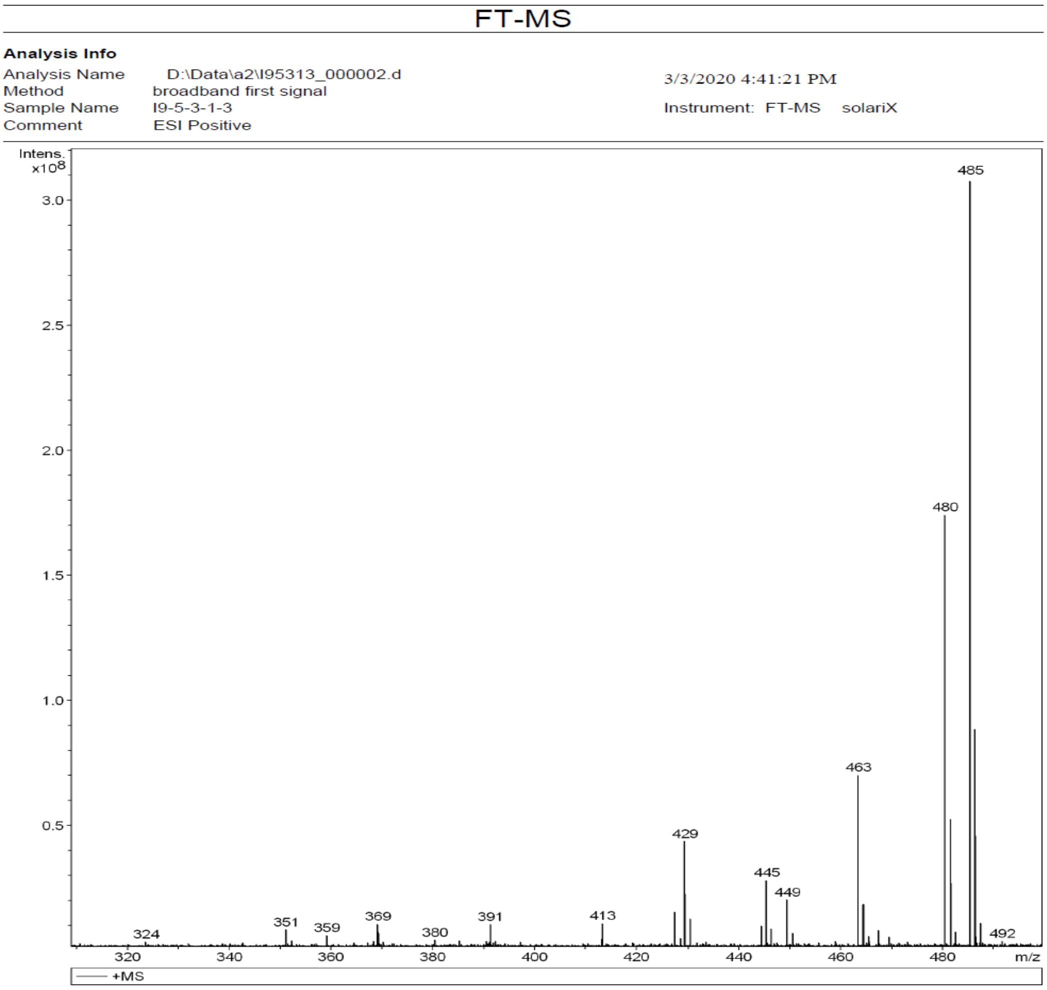

S20. ESIMS spectrum of compound **3**.

Mass Spectrum SmartFormula Report

Analysis Info

Analysis Name	D:\Data\a2\I95313_000003.d	3/12/2020 1:51:35 PM
Method	broadband first signal	Operator: YU HSIAO-CHING
Sample Name	I9-5-3-1-3	Instrument: BRUKER FT-MS solariX
Comment	ESI Positive	

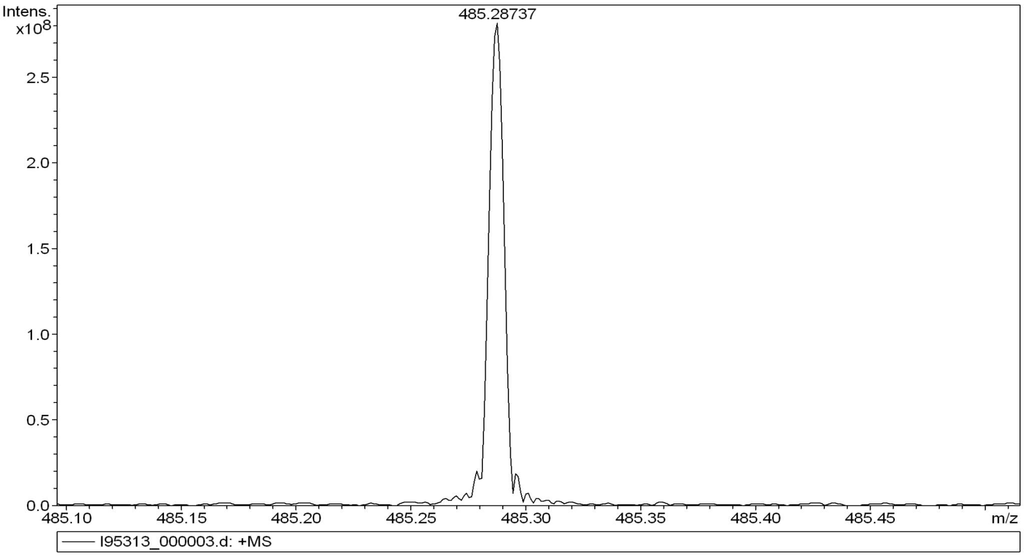

Meas. m/z	#	Formula	Score	m/z	err [mDa]	err [ppm]	mSigma	rdb	e⁻ Conf	N-Rule
485.28737	1	C 27 H 42 Na O 6	100.00	485.28736	-0.01	-0.03	11.7	6.5	even	ok

S21. HRESIMS spectrum of compound **3**.

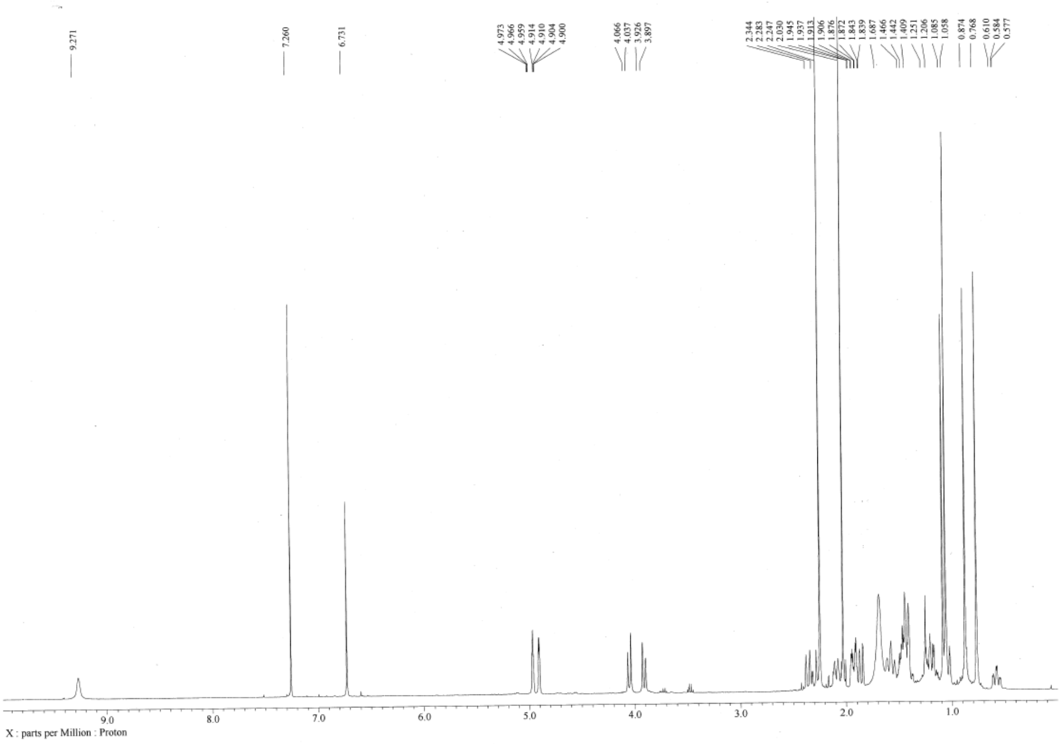

S22. ^{1}H NMR spectrum (400 MHz) of compound **3** in $CDCl_3$.

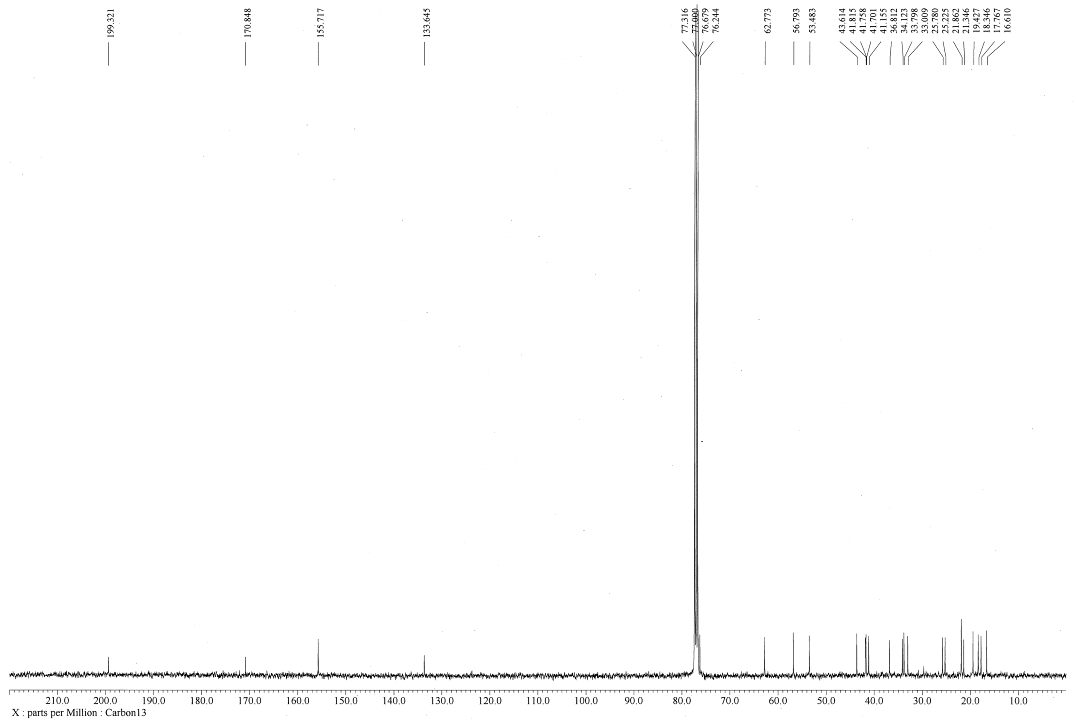

S23. ^{13}C NMR spectrum (100 MHz) of compound **3** in $CDCl_3$.

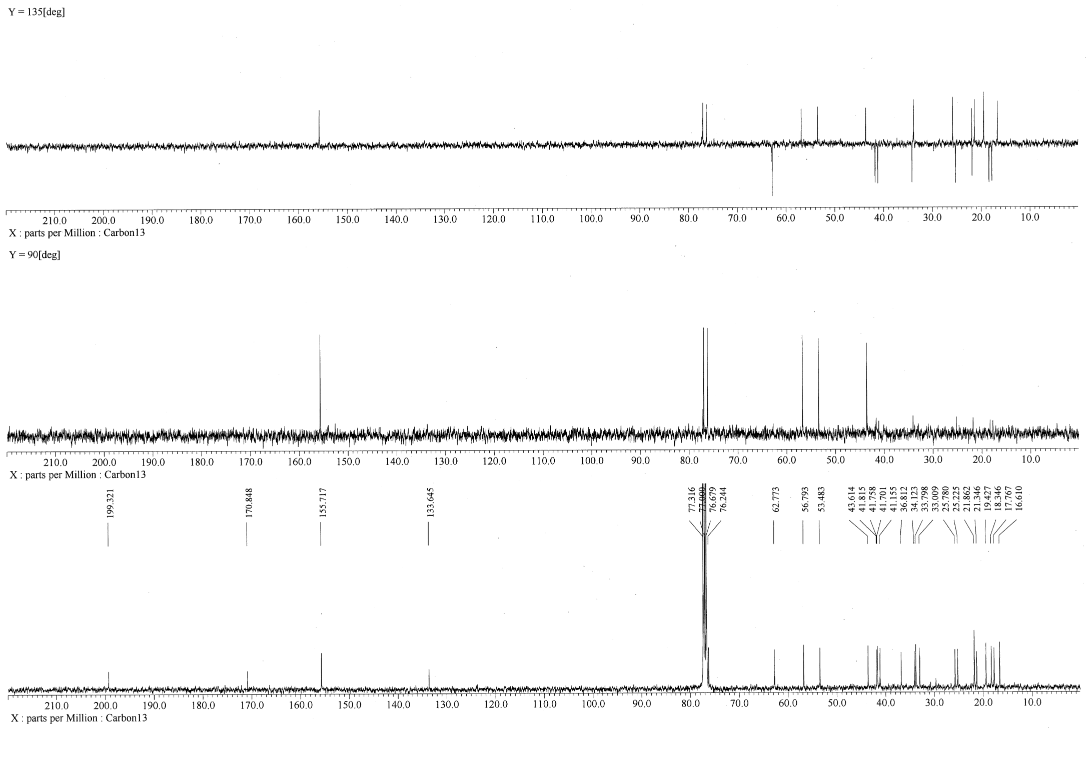

S24. DEPT spectrum of compound **3** in $CDCl_3$.

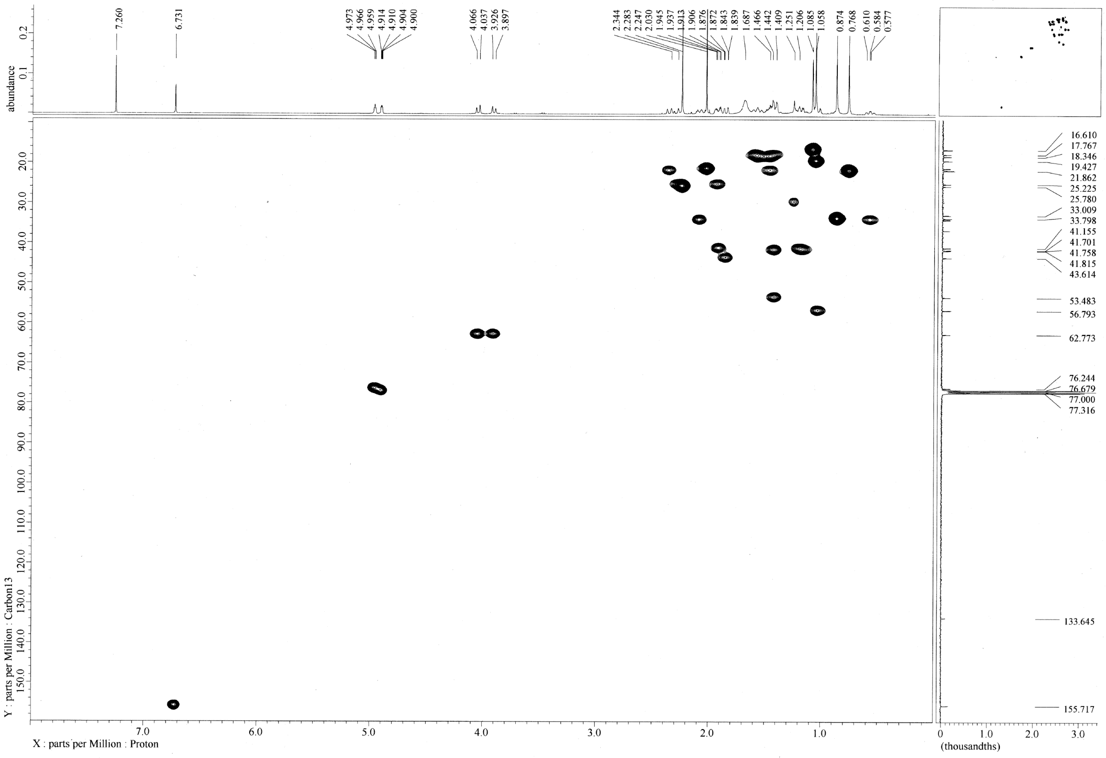

S25. HSQC spectrum of compound **3** in $CDCl_3$.

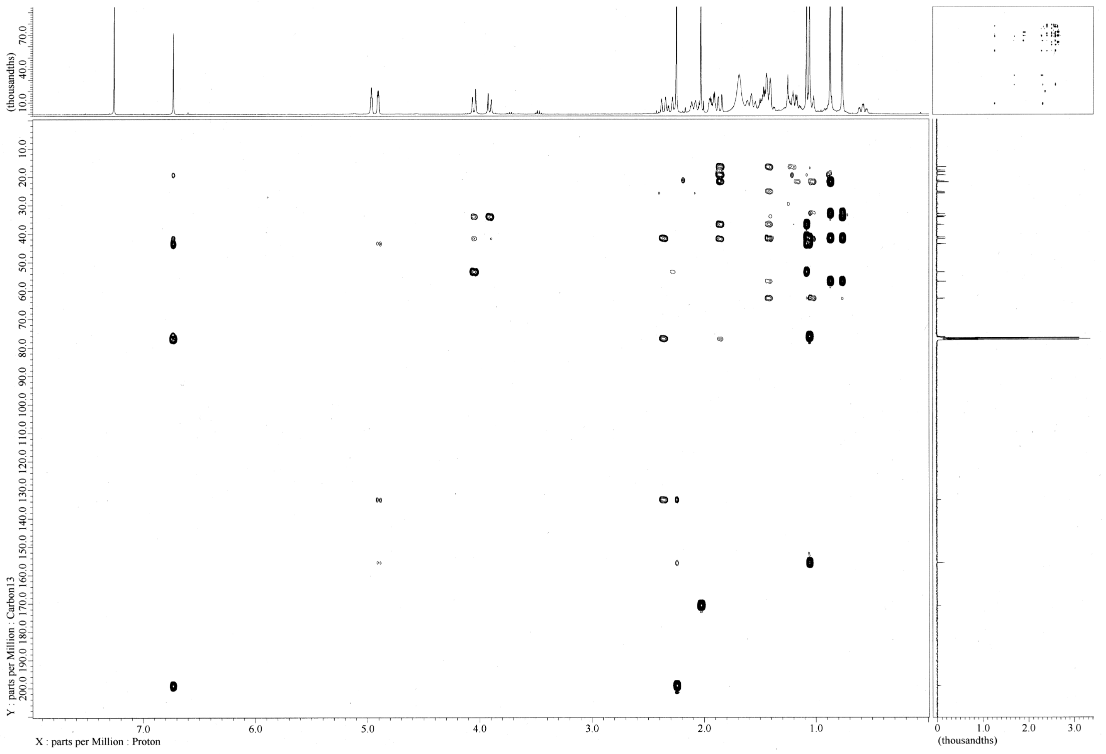

S26. HMBC spectrum of compound **3** in $CDCl_3$.

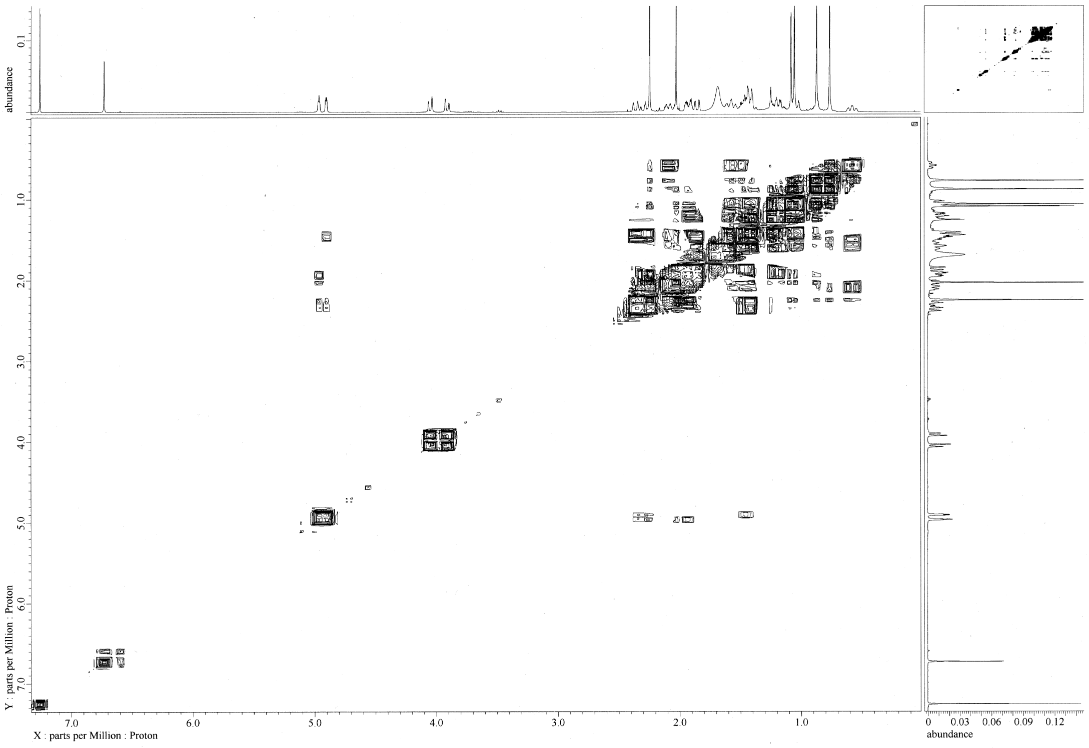

S27. 1H-1H COSY spectrum of compound **3** in $CDCl_3$.

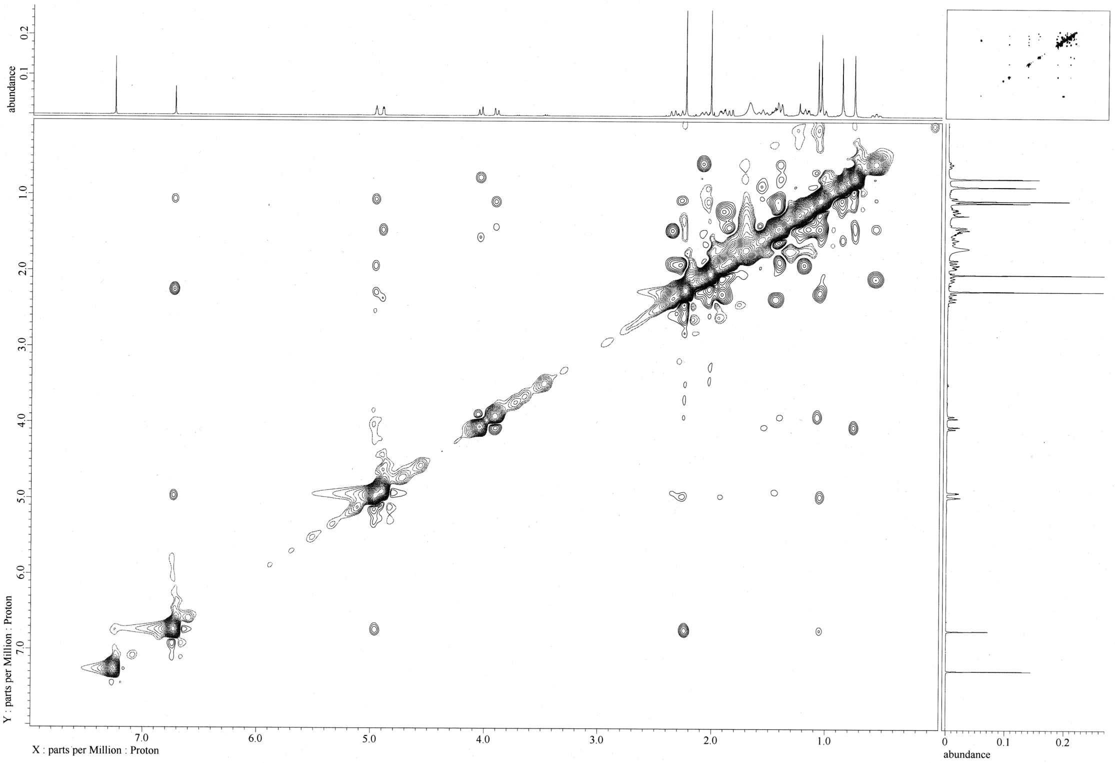

S28. NOESY spectrum of compound **3** in $CDCl_3$.

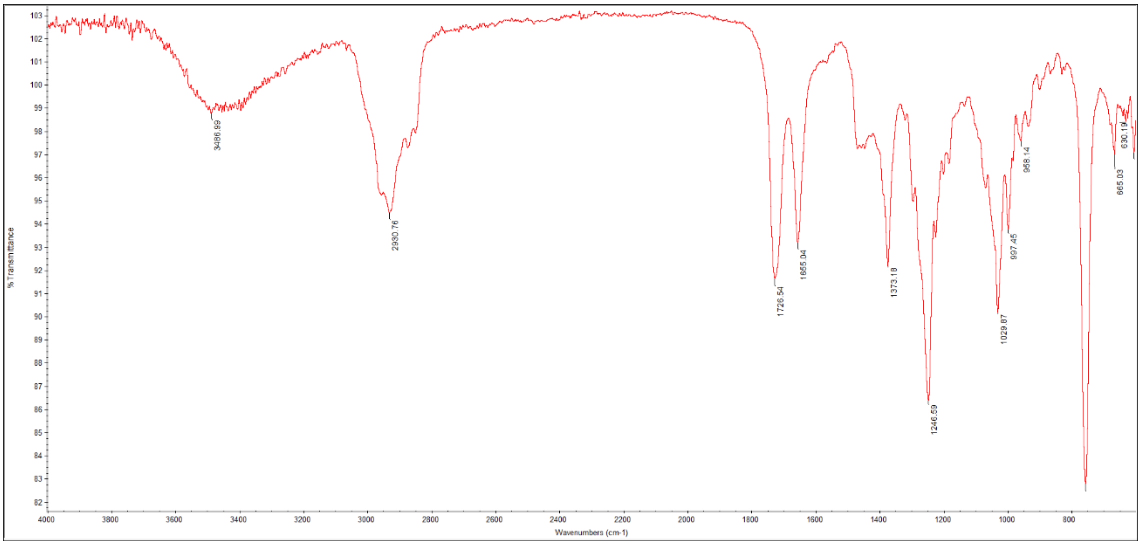

S29. IR spectrum of compound **4**.

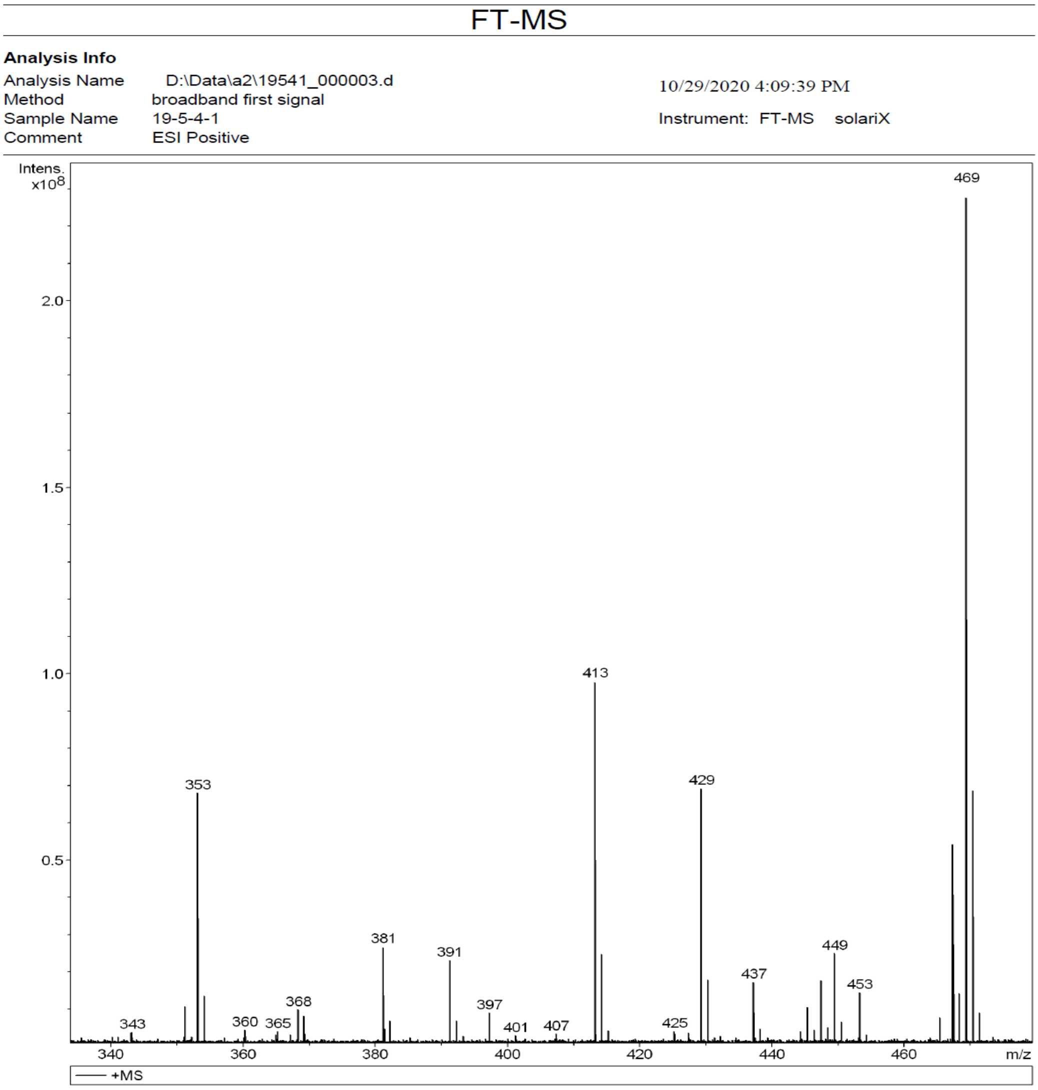

S30. ESIMS spectrum of compound **4**.

Mass Spectrum SmartFormula Report

Analysis Info

Analysis Name	D:\Data\a2\19541_000005.d	10/29/2020 4:12:48 PM
Method	broadband first signal	Operator: YU HSIAO-CHING
Sample Name	19-5-4-1	Instrument: BRUKER FT-MS solariX
Comment	ESI Positive	

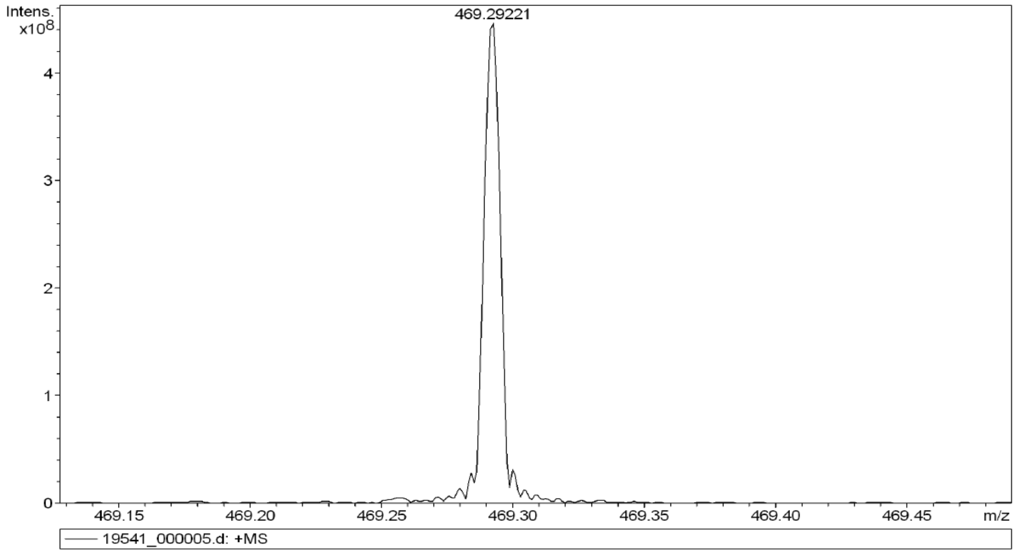

Meas. m/z	#	Formula	Score	m/z	err [mDa]	err [ppm]	mSigma	rdb	e⁻ Conf	N-Rule
469.29221	1	C 27 H 42 Na O 5	100.00	469.29245	0.24	0.50	10.3	6.5	even	ok

S31. HRESIMS spectrum of compound **4**.

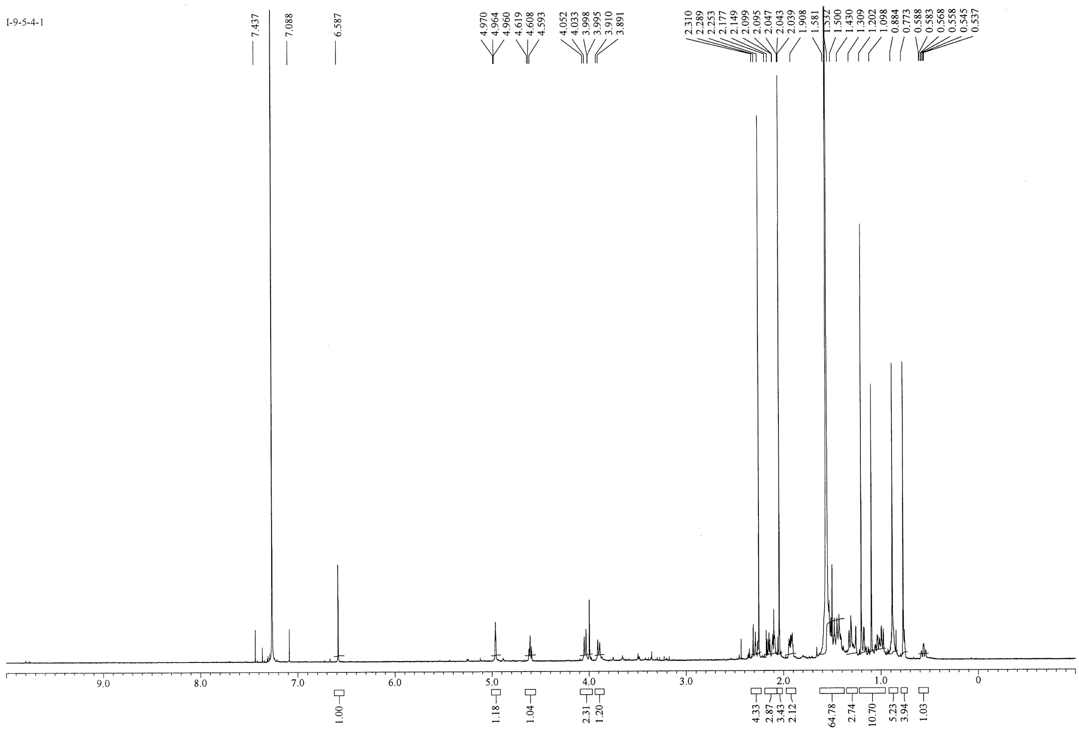

S32. ^{1}H NMR spectrum (600 MHz) of compound **4** in $CDCl_3$.

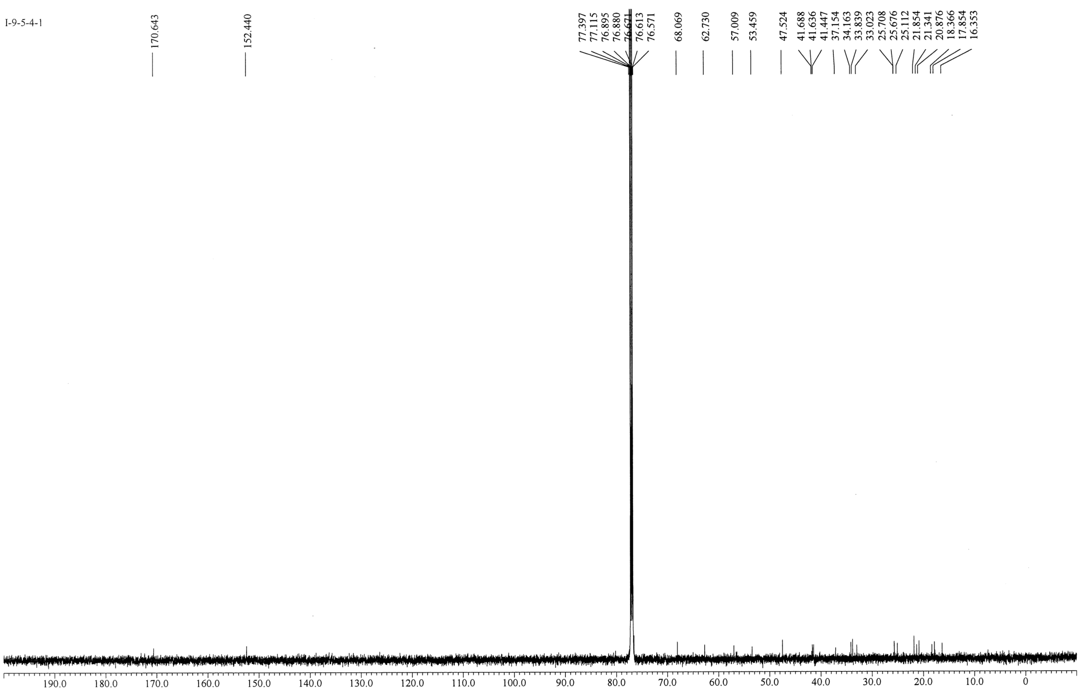

S33. ^{13}C NMR spectrum (150 MHz) of compound **4** in $CDCl_3$.

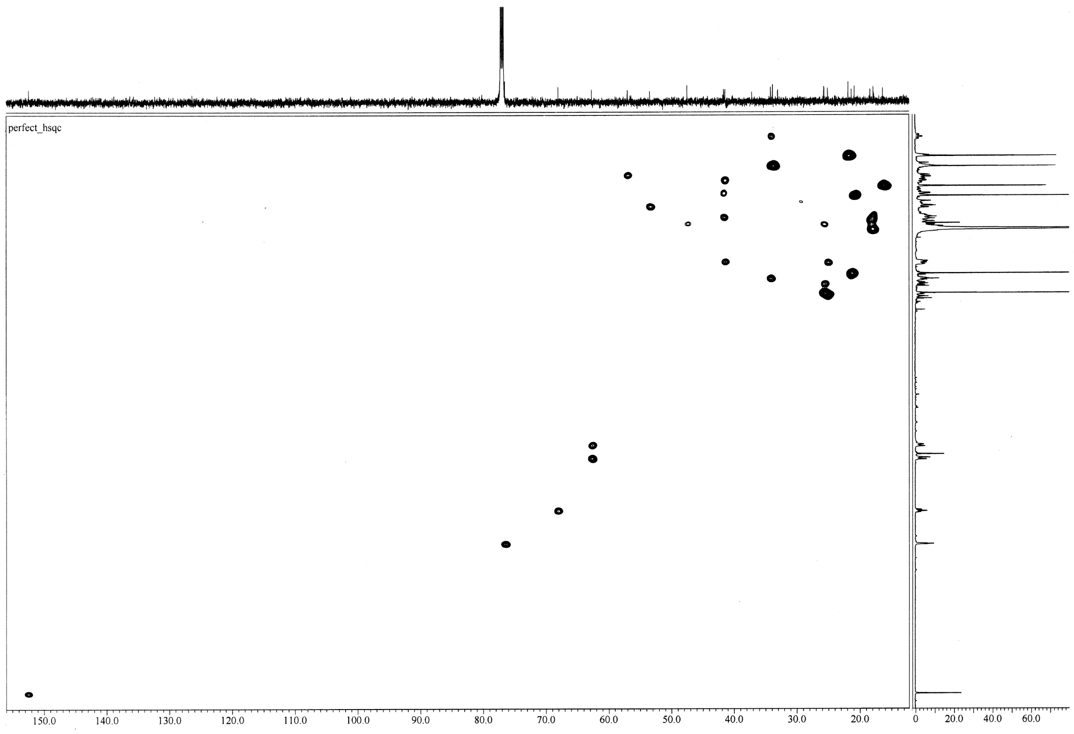

S34. HSQC spectrum of compound **4** in $CDCl_3$.

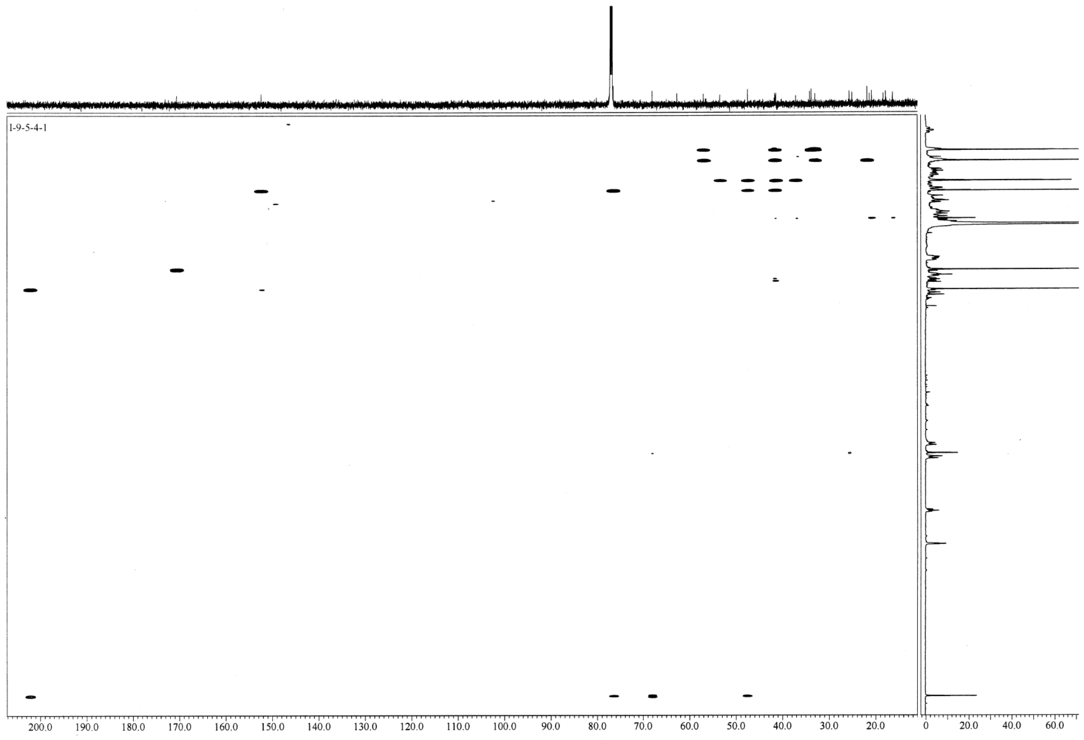

S35. HMBC spectrum of compound **4** in $CDCl_3$.

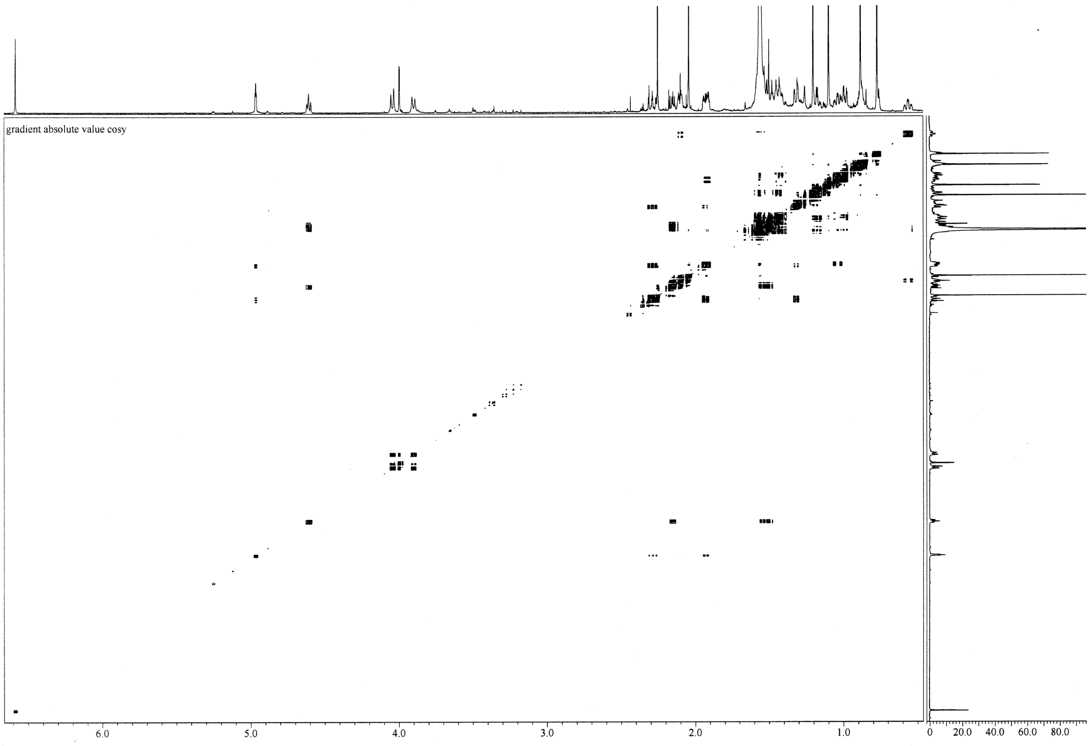

S36. ^1H-^1H COSY spectrum of compound **4** in $CDCl_3$.

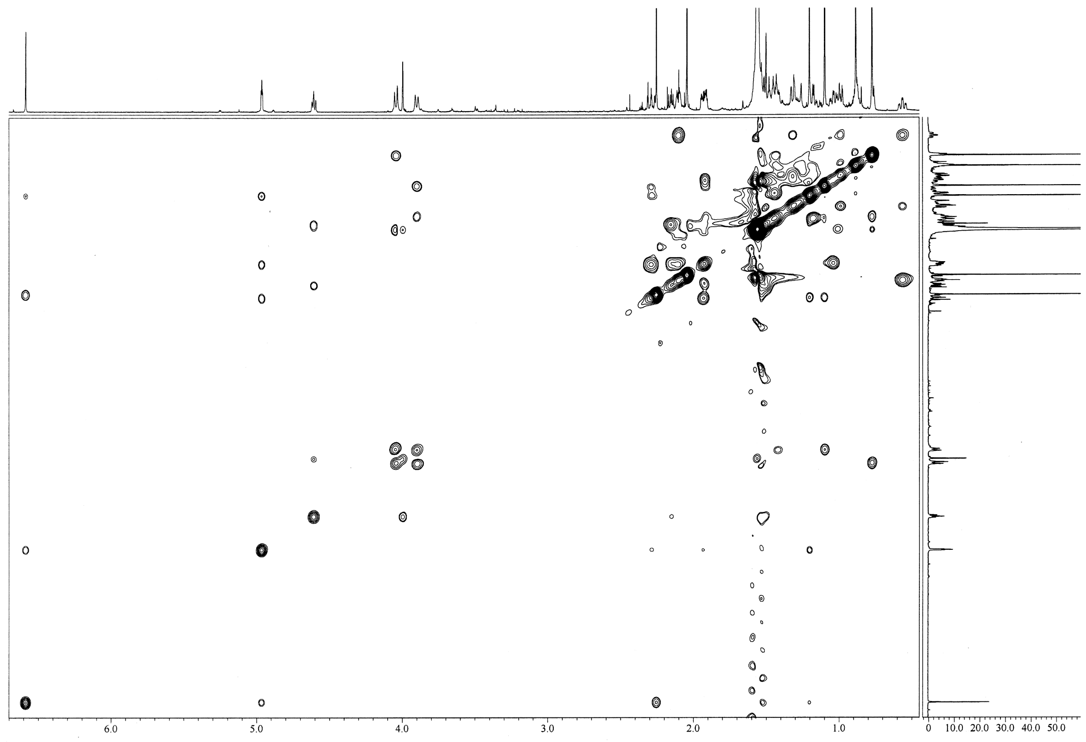

S37. NOESY spectrum of compound **4** in $CDCl_3$.
